# Germline *HOXB13* G84E mutation carriers and risk to twenty common types of cancer: results from the UK Biobank

**DOI:** 10.1038/s41416-020-01036-8

**Published:** 2020-08-24

**Authors:** Jun Wei, Zhuqing Shi, Rong Na, Chi-Hsiung Wang, W. Kyle Resurreccion, S. Lilly Zheng, Peter J. Hulick, Kathleen A. Cooney, Brian T. Helfand, William B. Isaacs, Jianfeng Xu

**Affiliations:** 1 Program for Personalized Cancer Care, NorthShore University HealthSystem, Evanston, IL USA; 2 Department of Medicine, NorthShore University HealthSystem, Evanston, IL USA; 3 Duke University School of Medicine and Duke Cancer Institute, Durham, NC USA; 4 Department of Urology and the James Buchanan Brady Urologic Institute, Johns Hopkins University School of Medicine, and the Sidney Kimmel Comprehensive Cancer Center at Johns Hopkins, Baltimore, MD USA

**Keywords:** Cancer genetics, Mutation, Genetic testing

## Abstract

Germline *HOXB13* G84E mutation has been consistently associated with prostate cancer (PCa) risk, but its association with other cancers is controversial. We systematically tested its association with the 20 most common cancer types in subjects from the UK Biobank. The G84E mutation was found in 1,545 (0.34%) of 460,224 participants of European ancestry. While mutation status did not associate with cancer risk in females, it was significantly associated with increased risk in males; odds ratio (OR) (95% confidence interval) for overall cancer diagnosis was 2.19 (1.89–2.52), *P* = 2.5E-19. The association remained after excluding PCa; OR = 1.4 (1.16–1.68), *P* = 0.003, suggesting association with other cancers. Indeed, suggestive novel associations were found for two other cancer types; rectosigmoid cancer, OR = 2.25 (1.05–4.15), *P* = 0.05 and non-melanoma skin cancer (NMSC), OR = 1.40 (1.12–1.74), *P* = 0.01. For NMSC, the association was found only in basal cell carcinoma, OR = 1.37 (1.07–1.74), *P* = 0.03. These findings have potential clinical utility for genetic counselling regarding *HOXB13*.

## Background

*HOXB13* was identified as a prostate cancer (PCa) susceptibility gene in 2012.^1^ A recurrent germline mutation (G84E) found exclusively in European descendants co-segregated with PCa diagnosis in families and presented at a significantly higher frequency in PCa patients (1.4%) compared to unaffected controls (0.1–0.4%) in the population. These findings have been consistently replicated in large published studies.^2,3^ In addition, suggestive evidence for associations between the G84E mutation and risk for leukaemia, bladder cancer, kidney cancer, non-Hodgkin’s lymphoma, and breast cancer was reported, although results were inconclusive due to small sample sizes of carriers.^4,5^ The objective of this study is to systematically assess the association of G84E mutation with risk for the 20 most common types of cancer in a large population-based study.

## Methods

Subjects of this study were from the UK Biobank (UKB), a population-based study with extensive genetic and phenotypic data for approximately 500,000 individuals from across the United Kingdom aged between 40 and 69 at recruitment (accessed under Application Number: 50295).^6^ Diagnosis of the 20 most common cancers, ranked by the 2018 GLOBOCAN database,^7^ were obtained from self-report, inpatient diagnosis, and the UK cancer registry. The G84E mutation (rs138213197) status was obtained from the UKB Axiom SNP genotype array. Odds ratio (OR) and 95% confidence interval (CI) were estimated separately in males and females using the logistic regression model adjusting for age at study recruitment and the top two principal components derived from 10,000 randomly selected SNPs across the genome.

## Results

A total of 1,545 *HOXB13* G84E mutation carriers were found in 460,224 self-reported European descendants (all heterozygotes), resulting in a carrier rate of 0.34%. The vast majority of carriers (73%) did not have a reported family history of cancer (family history among parents and siblings was reported for prostate, breast, colorectal, and lung cancer in the UKB). While the mutation was not associated with any cancer risk in females, it was significantly associated with overall cancer risk in males; OR (95% CI) was 2.19(1.89–2.52) for at least one type of cancer, *P* = 2.5E-19 (Table 1). The association remained after excluding PCa, OR = 1.4(1.16–1.68), *P* = 0.003, suggesting the mutation increases risk to other types of cancer besides of PCa.

**Table 1:** Association of the *HOXB13* G84E mutation and risk to common cancer in UK Biobank (White subjects only).

	Male, *N* = 210,354							Female, *N* = 249,870						
Type of cancer, ICD codes	No. of cases	No. of controls	No. (%) of G84E carriers in cases	No. (%) of G84E carriers in controls	OR (95% CI)^a^	*P*-value	Power^b^	No. of cases	No. of controls	No. (%) of G84E carriers in cases	No. (%) of G84E carriers in controls	OR (95% CI)^a^	*P*-value	Power^b^
Any type of cancer	34,137	176,217	206 (0.6%)	517 (0.29%)	2.19 (1.89–2.52)	2.95E-19	1.00	42,267	207,603	135 (0.32%)	687 (0.33%)	0.96 (0.82–1.12)	0.69	1.00
Any type of cancer, excluding PCa	26,008	176,217	100 (0.38%)	517 (0.29%)	1.39 (1.15–1.67)	3.00E-03	1.00	42,267	207,603	135 (0.32%)	687 (0.33%)	0.96 (0.82–1.12)	0.69	1.00
Specific type of cancer														
Oesophagus, C15	842	209,512	5 (0.59%)	718 (0.34%)	1.73 (0.74–3.35)	0.23	0.29	283	249,587	0 (0%)	822 (0.33%)	–	–	0.13
Stomach, C16	682	209,672	4 (0.59%)	719 (0.34%)	1.7 (0.65–3.54)	0.29	0.24	296	249,574	1 (0.34%)	821 (0.33%)	1.03 (0.11–3.79)	0.98	0.13
Colon, C18	2852	207,502	10 (0.35%)	713 (0.34%)	1.01 (0.57–1.65)	0.96	0.73	2313	247,557	6 (0.26%)	816 (0.33%)	0.79 (0.37–1.45)	0.56	0.64
Rectosigmoid, C19	780	209,574	6 (0.77%)	717 (0.34%)	2.24 (1.05–4.14)	0.05	0.27	486	249,384	1 (0.21%)	821 (0.33%)	0.62 (0.07–2.29)	0.64	0.19
Rectum, C20	1485	208,869	9 (0.61%)	714 (0.34%)	1.77 (0.96–2.95)	0.09	0.46	881	248,989	2 (0.23%)	820 (0.33%)	0.69 (0.16–1.84)	0.60	0.30
Liver, C22	464	209,890	2 (0.43%)	721 (0.34%)	1.25 (0.3–3.34)	0.75	0.18	313	249,557	2 (0.64%)	820 (0.33%)	1.97 (0.46–5.25)	0.34	0.14
Pancreas, C25	587	209,767	0 (0%)	723 (0.34%)	–	–	0.22	497	249,373	2 (0.40%)	820 (0.33%)	1.23 (0.29–3.29)	0.77	0.19
Lung, C34	1893	208,461	6 (0.32%)	717 (0.34%)	0.92 (0.43–1.69)	0.83	0.55	1,686	248,184	3 (0.18%)	819 (0.33%)	0.54 (0.17–1.23)	0.29	0.51
Melanoma of skin, C43	1855	208,499	3 (0.16%)	720 (0.35%)	0.47 (0.15–1.06)	0.19	0.55	2,339	247,531	5 (0.21%)	817 (0.33%)	0.65 (0.28–1.25)	0.33	0.64
Nonmelanoma of skin, C44	13,480	196,874	63 (0.47%)	660 (0.34%)	1.4 (1.11–1.73)	0.01	1.00	12,651	237,219	49 (0.39%)	773 (0.33%)	1.19 (0.93–1.51)	0.24	1.00
BCC, 8090-8098	10,823	199,531	50 (0.46%)	673 (0.34%)	1.37 (1.06–1.74)	0.03	1.00	10,352	239,518	37 (0.36%)	785 (0.33%)	1.09 (0.82–1.43)	0.60	1.00
SCC, 8070-8078	2059	208,295	6 (0.29%)	717 (0.34%)	0.84 (0.39–1.55)	0.68	0.59	1390	248,480	6 (0.43%)	816 (0.33%)	1.33 (0.62–2.44)	0.49	0.44
Breast, C50	–	–	–	–	–	–	–	16,864	233,006	52 (0.31%)	770 (0.33%)	0.93 (0.73–1.17)	0.63	1.00
Cervix uteri, C53	–	–	–	–	–	–	–	2154	247,716	5 (0.23%)	817 (0.33%)	0.70 (0.30–1.36)	0.43	0.61
Corpus uteri, C54	–	–	–	–	–	–	–	2410	247,460	5 (0.21%)	817 (0.33%)	0.63 (0.27–1.21)	0.30	0.66
Ovary, C56	–	–	–	–	–	–	–	1866	248,004	6 (0.32%)	816 (0.33%)	0.98 (0.46–1.79)	0.95	0.55
Prostate, C61	10,266	200,088	132 (1.29%)	591 (0.30%)	4.81 (4.06–5.68)	1.37E-53	1.00	–	–	–	–	–	–	
Kidney, C64	1115	209,239	4 (0.36%)	719 (0.34%)	1.04 (0.4–2.15)	0.94	0.36	650	249,220	2 (0.31%)	820 (0.33%)	0.94 (0.22–2.49)	0.93	0.23
Bladder, C67	2342	208,012	9 (0.38%)	714 (0.34%)	1.12 (0.61–1.86)	0.75	0.64	798	249,072	1 (0.13%)	821 (0.33%)	0.38 (0.04–1.40)	0.34	0.28
Brain, nervous system, C71/C72/C47	571	209,783	2 (0.35%)	721 (0.34%)	1.02 (0.24–2.71)	0.98	0.21	494	249,376	1 (0.20%)	821 (0.33%)	0.61 (0.06–2.24)	0.63	0.19
Thyroid, C73	192	210,162	1 (0.52%)	722 (0.34%)	1.52 (0.16–5.57)	0.68	0.10	608	249,262	1 (0.16%)	821 (0.33%)	0.50 (0.05–1.82)	0.49	0.22
Non-Hodgkin lymphoma, C85	1187	209,167	2 (0.17%)	721 (0.34%)	0.49 (0.12–1.29)	0.31	0.38	960	248,910	6 (0.62%)	816 (0.33%)	1.92 (0.90–3.53)	0.11	0.32

^a^OR (odds ratio) adjusted for age at study recruitment and the top two principal components.

^b^Statistical power to detect an association assuming G84E carrier rate = 0.34%, OR = 2 and significance level = 0.05.

When examining specific cancer types, we confirmed the association between the mutation and PCa risk [OR = 4.81 (4.06–5.68), *P* = 1.32E-53] and found two novel suggestive/weak/marginal associations. For non-melanoma skin cancer (NMSC), the male G84E carrier rate was 0.47% in 13,480 cases and 0.34% in 196,874 controls, OR = 1.40 (1.12–1.74), *P* = 0.01. Specifically, the association was found in basal cell carcinoma (BCC) [OR = 1.37 (1.07–1.74), *P* = 0.03] but not in squamous cell carcinoma (SCC) [OR = 0.84 (0.39–1.55), *P* = 0.68]. In comparison, the mutation was not significantly associated with BCC or SCC in females.

For rectosigmoid cancer, the male mutation carrier rate was 0.77% in 780 cases and 0.34% in 209,574 controls [OR = 2.25 (1.05–4.15), *P* = 0.05]. It is noted that the mutation had a higher but not statistically significant risk for rectal cancer [OR = 1.77 (0.96–2.95), *P* = 0.09] but not with more proximal colon cancers [OR = 1.02 (0.57–1.65), *P* = 0.96]. In females, the G84E mutation was not significantly associated with rectosigmoid, rectal, or colon cancer.

We also tested the association between G84E mutation and risk for developing multiple types of cancers concurrently. A significant association was found in males only; 5.39% of mutation carriers but 2.61% of non-carriers had two or more types of cancer [OR = 2.43 (1.75–3.36), *P* = 7.96E-08]. When the analysis was repeated after excluding PCa, NMSC and rectosigmoid cancer, no association was found [OR = 1.11 (0.41–2.97), *P* = 1.00]. These results further suggest the association of G84E mutation with multiple types of cancer is limited to these three types of cancer.

## Discussion

Our finding that the mutation is associated with risk to multiple types of cancer is consistent with a report from a large Kaiser Permanente cohort (*N* = 83,285) where the mutation was imputed (*r*^2^ = 0.57 with genotyped data).^5^ For specific types of cancer, we did not confirm previously reported higher carrier rates found in patients of bladder cancer,^4^ leukaemia,^4^ kidney cancer,^5^ non-Hodgkin’s lymphoma,^5^ and breast cancer.^5^ The number of mutation carriers were limited (<50) in each of these cancers.

A unique feature of this population-based study was that cancer cases were not selected based on clinical characteristics or family history; therefore, results are more applicable to the general population for developing broad genetic testing and counselling regarding *HOXB13*. The strong association of the G84E mutation and PCa risk (OR = 4.81) from this population-based study, together with the consistent finding from previously published studies, suggest this mutation be included in germline testing for assessing PCa risk for men in their thirties and forties. Men positive for this mutation should be considered as high risk for PCa, and this information should be considered by physicians and patients when discussing the need, timing, and frequency of PCa screening. The clinical implication of the G84E mutation on other types of cancer such as NMSC and rectosigmoid cancer, however, requires additional confirmation studies in independent study populations.

HOX genes encode homeodomain transcription factors that play a critical role in establishing the basic body plan during embryogenesis.^8^ *HOXB13* expression in the mouse embryo is highly restricted to the urogenital sinus (i.e. the anlage of the prostate and bladder), hindgut, and tailbud.^9^ In adult humans, *HOXB13* continues to be highly expressed in the prostate and to a lesser extent in the bladder, sigmoid and transverse colon, and skin (The Genotype-Tissue Expression [GTEx]). In the mouse foetus, *HOXB13* plays a role in cutaneous development and regeneration.^10^ Thus, several adult tissues which retain *HOXB13* expression appear to have an elevated risk of developing cancer in carriers of the G84E allele. Despite similar expression levels of *HOXB13* in the distal colon and skin in both sexes (GTEx), increased cancer risk was observed only in males. While it is tempting to speculate that androgens may sensitise tissues for G84E-associated carcinogenesis, the mechanism of this difference remains unknown.

Several limitations are noted. First, despite being the largest study on *HOXB13*, the number of G84E mutation carriers remains small in individuals with cancers other than prostate. Therefore, null associations should be interpreted with caution, especially for relatively uncommon types of cancer. The estimated power was limited for most of the cancers except NMSC, PCa (in male) and breast cancer (in female) (Table 1). Second, the age range of subjects in the UKB (40–69 years) may lead to under-estimated carrier frequency and OR if the mutation is associated with early deaths. Third, the reported *P*-values for the associations between the G84E mutation and risk to NMSC and rectosigmoid cancer were not corrected for multiple testing. The associations of the mutation with NMSC and rectosigmoid cancer were not statistically significant after adjusting for 42 multiple tests. Confirmation of these novel findings in an independent population is required.

## Conclusions

In conclusion, the *HOXB13* G84E mutation carrier rate is relatively common (0.34%) in European descendants. Male mutation carriers are clearly at increased risk for PCa, and possibly NMSC and rectosigmoid cancer, while no evidence of increased risk of any cancer was observed in females. These findings, if confirmed in other independent studies, may have potential clinical utility for genetic counselling.

## Data Availability

The data used in this study is available in the UK Biobank, a publicly available repository. Data was accessed through a Material Transfer Agreement under Application Reference Number: 50295. For additional information, please feel free to contact the corresponding author, Jianfeng Xu, DrPH.
